# Efficacy and safety of iruplinalkib (WX‑0593) on non‑small cell lung cancer with *SPECC1L‑ALK* fusion: A case report

**DOI:** 10.3892/etm.2023.12341

**Published:** 2023-12-05

**Authors:** Quan Zhang, Jialin Lv, Xi Li, Hui Zhang, Chenlin Zhu, Meng Wang, Meimei Si, Ying Hu, Shucai Zhang

**Affiliations:** 1 Department of Oncology, Beijing Chest Hospital, Capital Medical University, Beijing Tuberculosis and Thoracic Oncology Institute, Beijing 101149, P.R. China; 2 Department of Medical Affairs, Qilu Pharmaceutical Co., Ltd., Jinan, Shandong 250100, P.R. China; 3 Clinical Research Centre, Qilu Pharmaceutical Co., Ltd., Jinan, Shandong 250100, P.R. China

**Keywords:** non-small cell lung cancer, *ALK* fusion, tyrosine kinase inhibitor, iruplinalkib

## Abstract

The evidence of anaplastic lymphoma kinase (ALK) inhibitor for non-small cell lung cancer (NSCLC) harbouring sperm antigen with calponin homology and coiled-coil domains 1-like (*SPECC1L*)-*ALK* fusion was limited. In a previous case report, a Chinese, 44-year-old, female non-smoker with stage IV NSCLC harbouring *SPECC1L-ALK* fusion was treated with crizotinib ± bevacizumab for 23 months (from October 2017 to September 2019) and second-generation ALK inhibitor iruplinalkib for 2.5 months (from October 2019 to January 2020). The present study is an updated case report of subsequent follow-up of this patient. The patient participated in the phase II INTELLECT study and received iruplinalkib 180 mg once daily with a 7-day lead-in phase at 60 mg once daily. Systemic partial response was achieved 1 month later. Intracranial complete response was achieved nearly 5 months after iruplinalkib treatment initiation. Systemic and intracranial responses continued as of cut-off date (February 2023). The progression-free survival reached 39.3 months, with right censoring (progression did not occur during follow-up). Grade 3 hypertriglyceridaemia complicated with grade 2 hypercholesterolaemia recovered after fenofibrate treatment. The other adverse events were not noteworthy. Iruplinalkib demonstrated promising systemic and intracranial efficacy for NSCLC harbouring *SPECC1L-ALK* gene, with acceptable and manageable adverse events (for example, grade 3 hypertriglyceridaemia or grade 2 hypercholesterolaemia). Iruplinalkib may be an ideal option for patients with rare *ALK* fusions.

## Introduction

GLOBOCAN 2020 showed lung cancer is one of leading causes of cancer-associated mortalities (1.8 million mortalities worldwide and 710 thousand mortalities in China) (1). The majority of lung cancer is non-small cell lung cancer (NSCLC), accounting for 80-85% of cases (2). Anaplastic lymphoma kinase (*ALK*) fusion genes, which occur in 3-7% of patients with NSCLC, are important oncogenic drivers (3-5). Echinoderm microtubule associated protein-like 4-*ALK* was the first reported fusion gene in NSCLC (5) and TRK-fused gene-*ALK* and kinesin family member 5B-*ALK* have subsequently been found (6,7).

The first-generation ALK tyrosine kinase inhibitor (TKI) crizotinib is a potent targeted drug for patients with *ALK*-positive NSCLC (8). Nevertheless, most patients eventually develop progressive disease (PD). The main reason for crizotinib resistance is poor penetration into the central nervous system (9). Second-generation ALK TKIs, such as iruplinalkib (WX-0593), alectinib and brigatinib, can overcome crizotinib resistance (10-14).

In 2018, Dickson *et al* (15) first reported a rare alteration, sperm antigen with calponin homology and coiled-coil domains 1 like (*SPECC1L*)-*ALK* fusion, in epithelioid fibrous histiocytoma. Crizotinib can inhibit the proliferation and survival of *SPECC1L-ALK* transfected HEK293 cells *in vitro* (16), indicating ALK TKI may serve as a treatment option for NSCLC with *SPECC1L-ALK* fusion. However, there is a lack of clinical evidence. A NSCLC case with *SPECC1L-ALK* fusion, treated with crizotinib ± bevacizumab for 23 months and iruplinalkib for 2.5 months, has been previously reported (17). The patient maintained partial response (PR) as of the reporting date (January 2020). The present study reports subsequent follow-up of this case.

## Case report

Demographic and tumour characteristics and the course of treatment with crizotinib have been reported previously (17). In brief, the patient was a female non-smoker aged 44 years. The patient attended Beijing Chest Hospital (Beijing, China) for worsened cough and expectoration in August 2017. Stage IV lung adenocarcinoma with lymph node metastases and pleural effusion but no brain metastasis (cT4N3M1c) was diagnosed according to computed tomography (CT) and lung tissue and retroperitoneal lymph node puncture biopsy. Lung tissue was tested using immunohistochemistry (IHC; Ventana ALK D5F3 CDx Assay, Roche), showing positive ALK expression (17). Further, next-generation sequencing was performed, and *SPECC1L-ALK* fusion was identified with a mutant allele frequency (MAF) of 28.66% (17). In October 2017, crizotinib treatment (250 mg, orally, twice daily) was initiated. In December 2017, PR was achieved according to Response Evaluation Criteria in Solid Tumors version 1.1 (RECIST v1.1) (18). In March 2018, bevacizumab was added to the regimen because tumour size increased and stable disease was reached. In September 2018, the patient developed PD, with progression-free survival (PFS) of nearly 23 months on crizotinib ± bevacizumab. A second biopsy indicated increased ALK expression in IHC and the same *SPECC1L-ALK* fusion with a lower MAF (1.5%) (17). In October 2019, combination of crizotinib and bevacizumab was discontinued.

The patient participated in the single-arm phase II INTELLECT study of iruplinalkib (10) at Beijing Chest Hospital in October 2019. In the INTELLECT study, patients with *ALK*-positive crizotinib-resistant advanced NSCLC aged ≥18 years with Eastern Cooperative Oncology Group performance status (19) of 0-2 were included. The primary endpoint was the independent review committee-assessed objective response rate. At baseline, the patient had lymph node and brain metastases and pleural effusion. The brain metastasis was first found at screening of the INTELLECT study. According to the protocol of the INTELLECT study, oral iruplinalkib 180 mg once daily with a 7-day lead-in phase at 60 mg once daily was given. Tumour was assessed every 6 weeks in the first 24 weeks after iruplinalkib treatment initiation, every 9 weeks after 24 weeks and every 12 weeks after 1 year until disease progression, withdrawal of consent, loss of follow-up, start of other antitumor treatment or death. For this patient, chest and upper abdomen CT and brain magnetic resonance imaging were used. Systemic and intracranial response was evaluated according to RECIST v1.1 and Response Assessment in Neuro-Oncology (RANO) brain metastases criteria (20), respectively. Adverse events were classified and graded according to National Cancer Institute Common Terminology Criteria for Adverse Events, version 4.03(21).

**Figure 1:**
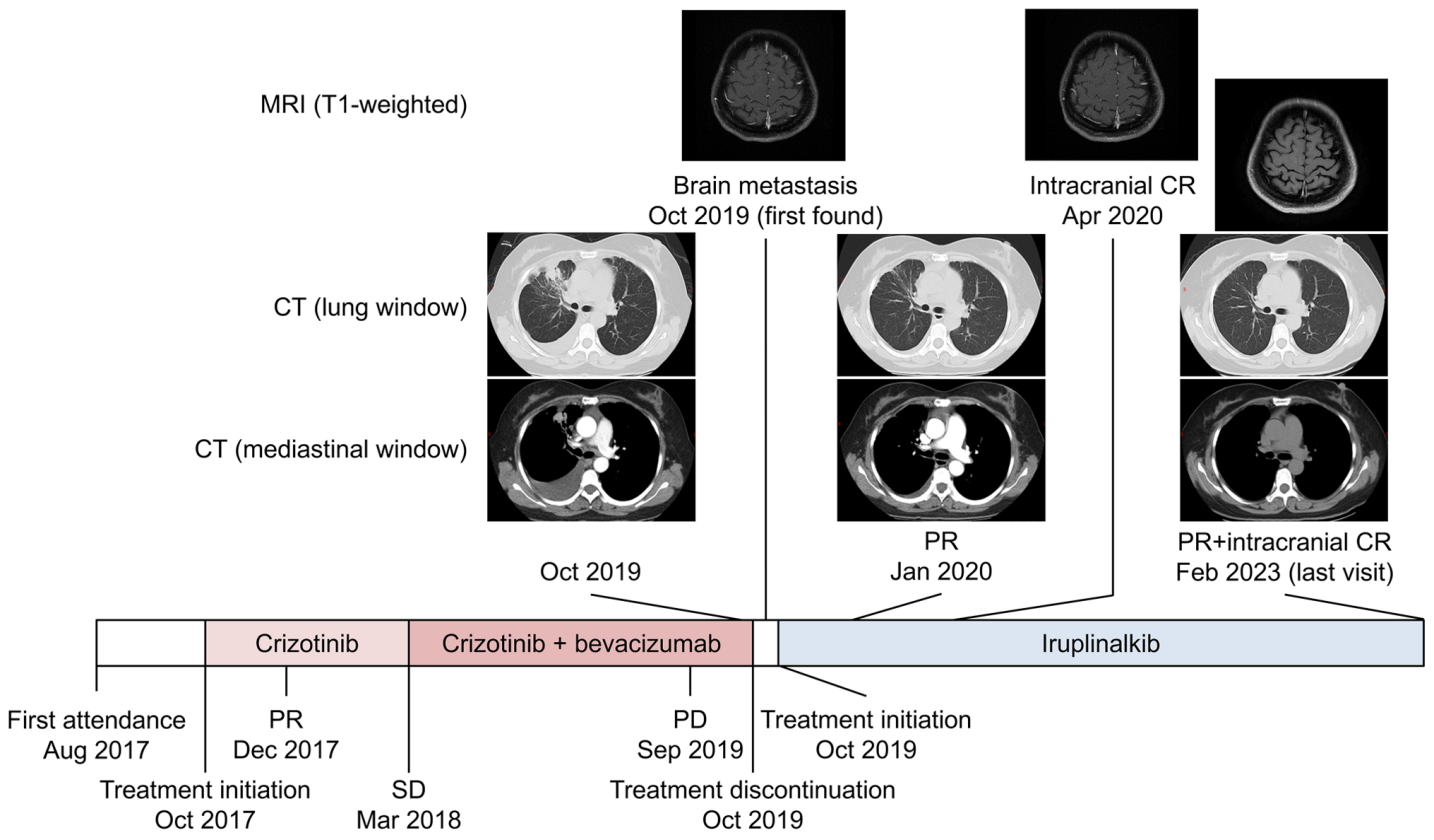
Treatment process and computed tomography images. MRI, magnetic resonance imaging. CR, complete response; CT, computed tomography; PR, partial response; SD, stable disease; PD, progressive disease.

PR was achieved one month later (November 2019). This patient was followed-up at 4th week but not 6th week. Additionally, the intracranial lesion disappeared and intracranial complete response was achieved in April 2020. As of February 2023, systemic PR and intracranial complete response were maintained (Fig. 1). The PFS on iruplinalkib lasted for 39.3 months, with right censoring.

No notable treatment-related adverse event (TRAE) was observed except grade 3 hypertriglyceridaemia complicated with grade 2 hypercholesterolaemia. These TRAEs were first reported in November 2019. Oral fenofibrate 200 mg twice daily was given and triglyceride and cholesterol levels reverted to baseline values (Table I). There was no dose interruption, reduction or discontinuation due to TRAE.

## Discussion

*SPECC1L-ALK* fusion is a rare alteration first reported in epithelioid fibrous histiocytoma in 2018(15). In 2020 and 2021, this fusion was identified in NSCLC and glioma, respectively (17,22). The present study reported subsequent follow-up of a patient with NSCLC harbouring *SPECC1L-ALK*.

Generally, as oncogenic drivers, ALK fusion proteins can result in continuous ALK and downstream signalling pathway activation, inducing tumour proliferation and survival (23). However, the mechanism of SPECC1L-ALK fusion proteins in ALK and downstream signalling pathway activation is unclear. ALK TKIs are recommended targeted drugs for *ALK*-positive disease (8). *In vitro*, crizotinib, a first-generation ALK TKI, inhibits growth and survival of *SPECC1L-ALK* transfected HEK293 cells in a dose-dependent manner (16). Nevertheless, to the best of our knowledge, there is a lack of studies of novel ALK TKIs for patients with NSCLC and *SPECC1L-ALK*.

The present case received crizotinib as the first-line treatment and achieved PR. Then, bevacizumab was added due to increased tumour size. First-line PFS reached ~23 months. After PD on crizotinib + bevacizumab, second-generation ALK TKIs may be ideal treatment options (10-14,24-26).

Iruplinalkib is a second-generation ALK TKI developed by Qilu Pharmaceutical Co., Ltd. (Jinan, China) with potent preclinical and clinical efficacy and acceptable safety profiles in *ALK*-positive NSCLC (10,27-29). The phase II INTELLECT study (10) of iruplinalkib showed an objective response rate and a PFS superior or similar to other second-generation ALK TKIs (for example, ceritinib and brigatinib) (11-14,24-26). The present patient had systemic PR and intracranial CR, which continued as of cut-off date (February 2023). The PFS was 39.3 months with right censoring, notably longer than the investigator-assessed median PFS of 14.5 months in the INTELLECT study. In terms of TRAE, most were grade 1 to 2 and not notable in the present patient. Grade 3 hypertriglyceridaemia and grade 2 hypercholesterolaemia occurred during iruplinalkib treatment. Triglyceride and cholesterol levels reverted to baseline after oral fenofibrate 200 mg twice daily was administered. No treatment-related dose interruption, reduction or discontinuation occurred.

In summary, iruplinalkib had promising systemic and intracranial efficacy for NSCLC harbouring *SPECC1L-ALK* gene, with acceptable and manageable safety profiles. Iruplinalkib may be used for patients with rare *ALK* fusions. The oncogenic mechanisms of SPECC1L-ALK fusion protein and anti-tumour activity of ALK TKI on tumour with *SPECC1L-ALK* need further investigation.

## Figures and Tables

**Table I:** TRAEs.

TRAE	Time of occurrence after treatment initiation, days	Grade	Management	Duration, days	Outcome
Nausea	8	1	Observation	3	Recovered without sequelae
Vomiting	8	1	Observation	3	Recovered without sequelae
Diarrhoea	10	1	Observation	3	Recovered without sequelae
Hypercholesterolaemia	10	2	Fenofibrate (200 mg, orally, twice daily)	156	Recovered without sequelae
Hypertriglyceridaemia	10	3	Fenofibrate (200 mg, orally, twice daily)	444	Recovered without sequelae
Hypertension	13	2	Fosinopril (10 mg, orally, once daily) Then switched to nifedipine (30 mg, orally, once daily) and irbesartan and hydrochlorothiazide (150 mg/12.5 mg, orally, once daily)	Up to cut-off date (February-2023)	Not recovered
Hepatic function abnormal	20	1	Bicyclol (25 mg, orally, thrice daily)	42	Recovered without sequelae
Anaemia	116	1	Observation	50	Recovered without sequelae
Hyperuricaemia	1,028	1	Observation	Up to cut-off date (February-2023)	Not recovered

TRAEs were classified and graded according to the National Cancer Institute Common Terminology Criteria for Adverse Events, version 4.03. TRAE, treatment-related adverse event.

## Data Availability

The datasets used and/or analysed during the current study are available from the corresponding author on reasonable request.
